# Effects of Externally Applied Electric Fields on the Manipulation of Solvated-Chignolin Folding: Static- versus Alternating-Field Dichotomy at Play

**DOI:** 10.1021/acs.jpcb.1c06857

**Published:** 2022-01-10

**Authors:** HaoLun Wu, Mohammad Reza Ghaani, Zdeněk Futera, Niall J. English

**Affiliations:** † School of Chemical & Bioprocess Engineering, University College Dublin, Belfield, Dublin 4, Ireland; ‡ Faculty of Science, University of South Bohemia, České Budějovice 370 05, Czech Republic

## Abstract

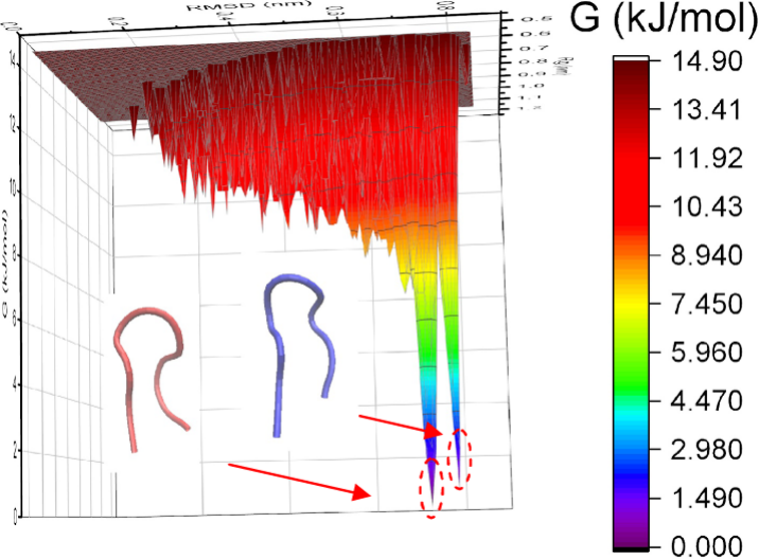

The interaction between a protein and external electric field (EF) can alter its structure and dynamical behavior, which has a potential impact on the biological function of proteins and cause uncertain health consequences. Conversely, the application of EFs of judiciously selected intensity and frequency can help to treat disease, and optimization of this requires a greater understanding of EF-induced effects underpinning basic protein biophysics. In the present study, chignolin—an artificial protein sufficiently small to undergo fast-folding events and transitions—was selected as an ideal prototype to investigate how, and to what extent, externally applied electric fields may manipulate or influence protein-folding phenomena. Nonequilibrium molecular dynamics (NEMD) simulations have been performed of solvated chignolin to determine the distribution of folding states and their underlying transition dynamics, in the absence and presence of externally applied electric fields (both static and alternating); a key focus has been to ascertain how folding pathways are altered in an athermal sense by external fields. Compared to zero-field conditions, a dramatically different—indeed, bifurcated—behavior of chignolin-folding processes emerges between static- and alternating-field scenarios, especially vis-à-vis incipient stages of hydrophobic-core formation: in alternating fields, fold-state populations diversified, with an attendant acceleration of state-hopping folding kinetics, featuring the concomitant emergence of a new, quasi-stable structure compared to the native structure, in field-shifted energy landscapes.

## Introduction

Proteins induce structural transitions to activate biological functions, such as ligand binding and release, catalysis, and signal transduction.^1^ To elucidate further the underlying nature of these important functional phenomena, clarifying the underlying mechanisms of structural transitions—folding—is indispensable.^1^ As one of the most fundamental and fascinating biological processes, protein folding remains a long-standing conundrum in our understanding of the detailed mechanisms of how protein structure emerges from primary sequence to native three-dimensional structures.^2^ Although a typical protein has numerous conformations, which makes a thorough search in determining its native structure in its global-minimum free-energy state a formidable challenge, protein-folding events per se between states are known to be relatively rapid processes. This is in contrast to the waiting, or dwell, times in particular configurations in between these interstate transitions, which can often be lengthy, and are subject to randomness and statistical distributions.^3^ Indeed, the characterization of the folding dynamics, together with the free-energy landscape (FEL) of proteins’ myriad of complex structural states, constitutes an important puzzle—not only in understanding the protein-folding mechanism in and of itself, but also in solving the above-mentioned “paradox” of bifurcated, “long-short” state-to-state transition kinetics, with often long configuration-residence times punctuated fluidly by typically rapid triggered/random “state shifts”.^3^

The study of the effects of externally applied electric field (EF) on the properties of organic and inorganic materials has attracted widespread attention^4−10^—particularly in the guise of thermal effects. In particular, the function of proteins depends directly on their structure and any stress imposed by the presence of electric and/or electromagnetic (e/m) fields can potentially become harmful. For example, various amyloidopathies, such as Creutzfeldt–Jakob, Alzheimer’s patients, and Parkinson’s diseases, have been associated with protein conformational changes.^11^ In addition, there is evidence that microwave radiation may speed up the rates of folding and unfolding of globular proteins in solution.^12^ Microwave radiation has been shown to enhance the aggregation of bovine serum albumin in vitro without bulk heating,^7^ and exposure to electric and e/m fields can be considered in the design of alternative treatment strategies for amyloid diseases, due to their inhibitory effect on intermediate-strength amyloid-genic peptide conformations.^13^ Indeed, these results point out the importance of studies focusing on the alterations of protein conformations under the influence of e/m fields,^11^ with particular emphasis on the role of hydrogen bonds.^11,12^ Hydrogen bonds play a fundamental role in controlling the activity of proteins during enzyme action—folding, binding with other proteins, and other processes. Changes in the strength of hydrogen bonds, induced by an electric field, may affect these processes.

Despite the variety of proposed field-affected processes (e.g., chemical binding, ion transport, receptor function, protein conformation) and the different biophysical models used (e.g., anomalous energy diffusion, nonlinear dynamics, molecular resonance, electron tunneling, dipole interactions), a complete understanding of the observed—and often conflicting—experimental evidence of electric-field effects is still lacking.^14−16^ The main reason is the enormous complexity of the biological systems per se, which hinders the accurate estimation of the nature and the extent of electric- and e/m-field exposure to living organisms.

Understandably, to overcome the inherent complexity of multidimensional proteins, there is a desire to simplify the “experimental design”, with a view to isolating field effects more accurately—i.e., to disentangle and divorce, insofar as possible, field effects from the implicit, complex-system dynamics of larger proteins. With that in mind, we turn to chignolin, as one of the smallest, viable alternatives for classical definition as a protein, and opt here to study external-field effects thereon. Chignolin is an artificial peptide that forms a stable β-hairpin in water.^17^ Although it is composed of only 10 residues, it meets the requirements of protein categorization—folding into a unique structure and having a cooperative thermal transition between its unfolded and folded states.^18^ Previous theories state that folding begins with the formation of a turn or hydrophobic collapse.^5,19−21^ In the final stage of folding, the formation of the native hydrogen-bond network and the hydrophobic core, as well as the arrangement of side-chain packing occur simultaneously^3^—and we are motivated by consideration of these questions in the context of field-altered thermodynamic landscapes and kinetics in the case of solvated chignolin. In addition, chignolin is believed to fold into a β-hairpin on timescales ranging from nano- to microseconds because of its small size and high extent of “foldability”, or floppiness, irrespective of the surrounding molecular milieux.^17^ Simulations of chignolin, therefore, are expected to provide us with essentially complete information about its folding dynamics at the atomistic level. We note with interest that ref (22) has shown that a static external electric field interacting with chignolin’s dipole moment stabilizes its orientation and restricts its flexibility; for weaker applied fields, this has mixed effects on the creation, destruction, or indeed the strength of hydrogen bonds. However, under the influence of strong fields, chignolin unfolding is initiated by the separation of its terminal residues.^22^

Molecular dynamics (MD) has been used widely to explore, among other things, the role of flexibility in ligand binding, to study the rapid solvation of the electron transfer state in photosynthesis, to determine protein structures from NMR, and to calculate free-energy changes resulting from mutations in proteins.^23^ However, comparatively few MD studies have focused on the nonthermal effects of e/m fields on proteins; what limited work has been carried out in such a vein has focused more especially on the induced conformational changes in lysozyme, amyloid fibrils, and trans-membrane proteins.^11,24−26^ These studies were motivated by experimental evidence of e/m effects on protein denaturation, stability, and activity. In the longer run, similar studies can be used for electric- and e/m-field-based protein engineering for, say, clinical applications which hinge upon—indeed, exploit—the close relationship between proteins’ function and structure. Previous MD studies on oscillating-field effects on protein structures relied upon relatively complex molecules, mainly featuring helical secondary structures (e.g., insulin, lysozyme).^8,27^ In addition, reported MD simulation lengths for chignolin have been no longer than 1 μs^3,5,18,28,29^—during which many molecular behaviors and, indeed, state-to-state transitions do often tend to lack repeatability, and, by extension, the opportunity for closer statistical and mathematical scrutiny.

Therefore, in the present study, we have chosen a single solvated, chignolin molecule as an ideal protein-prototyping “case in point”—a simplified “playground” in which we can distill and dissect the essence of static- and oscillating-field effects on it through nonequilibrium molecular dynamics (NEMD) simulation, while disentangling as much as possible (lower-intensity) external-field coupling to inherently complicated underlying dynamics—which is undoubtedly present in more complex, larger proteins. At the same time, we extend MD trajectories in the absence and presence of such external fields into the multi-microsecond régime, to sample, with deterministic MD, a greater number of state-to-state transitions by natural, Boltzmann thermal statistical sampling, and also to enable more sophisticated kinetic modeling from various statistical perspectives. Furthermore, leveraging such longer-time, lower-field-strength NEMD simulations, we construct field-manipulated/shifted free-energy landscapes and Ramachandran plots to help characterize more subtly both static- and e/m-field effects on the thermodynamics properties of out-of-equilibrium chignolin folding.

## Methodology

The starting structure for the simulations of chignolin molecule was the mutant conformation CLN025 (Protein Data Bank (PDB) accession code 5AWL(30)), in which two terminal glycine residues were replaced by tyrosine separately, and this optimized structure is more thermodynamically stable and undergoes faster folding than the original chignolin structure (PDB accession code 1UAO(17)). All NEMD simulations for the chignolin in water solution were performed using the GPU version of GROMACS 2018^31^ simulation package, in conjunction with the AMBER99SB force field^32^ and TIP4P2005^33^ water model. As shown in ref (34), chignolin was highly stable in all AMBER99SB simulations, where both increased unfolding and decreased refolding free-energy barriers contributed to significantly higher stability of the chignolin native structure. As a charge-distribution-optimized model, TIP4P2005 presents excellent accuracy with respect to electrostatics and dynamic properties, as well as for sodium chloride solutions.^35^ Initial protein structures in unfolded straight configurations were formed by pulling the gas-phase structure by C- and N-termini, for unbiased observation of the folding process. For the overall, electroneutral system, the net charge of −2 *e* for the protein (Asp3, Glu5) was compensated by the NaCl solution (normal saline concentration 9 g/L equal to 1 NaCl/360 water molecules), where 10 Na^+^ and 8 Cl^–^ were placed in the system, together with 3600 water molecules. The protein was placed at the center of a cubic periodic box of size 49.67 Å, in the laboratory Cartesian frame of the original structure, which was sufficiently large to avoid any interactions with its periodic image replicas.

A 5 μs NPT-NEMD simulation was performed at 300 K and 1 bar using the modified Berendsen thermostat and Parrinello–Rahman methods,^31^ and the coordinates were saved every 1 ps for analysis. All bonds involving hydrogen were constrained using the LINCS method, and both nonbonded and long-range electrostatic interactions were handled by the particle-mesh Ewald (PME) method with a cutoff and integration time step of 10 Å and 2 fs, offering excellent Hamiltonian conservation. During the simulations, uniform external electric fields (EF) were applied to the system along the laboratory *x*-direction and classical mechanics was used for the treatment of the EF absorption since the experimental spectrum of liquid water is continuous in the low-frequency microwave region.^36^ The total forces acting on each partial charge were modeled according to Newton’s equation of motion
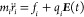
1where *f*_*i*_ is the molecular mechanics interatomic interactions force on site *i* based on the selected force field, *q*_*i*_ denotes the charge, and the electric vector ***E***(*t*) = *E*_max_ cos(ω*t*)***i*** has been discussed in detail in previous studies.^37−39^ In the present study, the folding process of chignolin was investigated under zero-field, static electric field (0.02 V/Å) and oscillating electric field (root mean square (r.m.s.) 0.02 V/Å, 2.45 GHz). The 0.02 V/Å external-field (r.m.s.) strength is lower than that in ref (22), well within the system’s linear-response régime, and is also of the order of 1% of intrinsic electric field intensities.^26^ Although it is, admittedly, somewhat above the dielectric-breakdown level in water (about 0.006 V/Å), this owes to a need to observe a tangible signal-to-noise ratio, even within the context of multi-microsecond NEMD, given that the underlying molecular dynamics is still, nevertheless, deterministic (to afford, ipso facto, realistic out-of-equilibrium system dynamics). In any event, the nondissociable nature of the presently used molecular mechanics force fields renders this a rather “moot point”.

## Results and Discussion

### Root-Mean-Square Deviation (RMSD) and State Analysis

**Figure 1:**
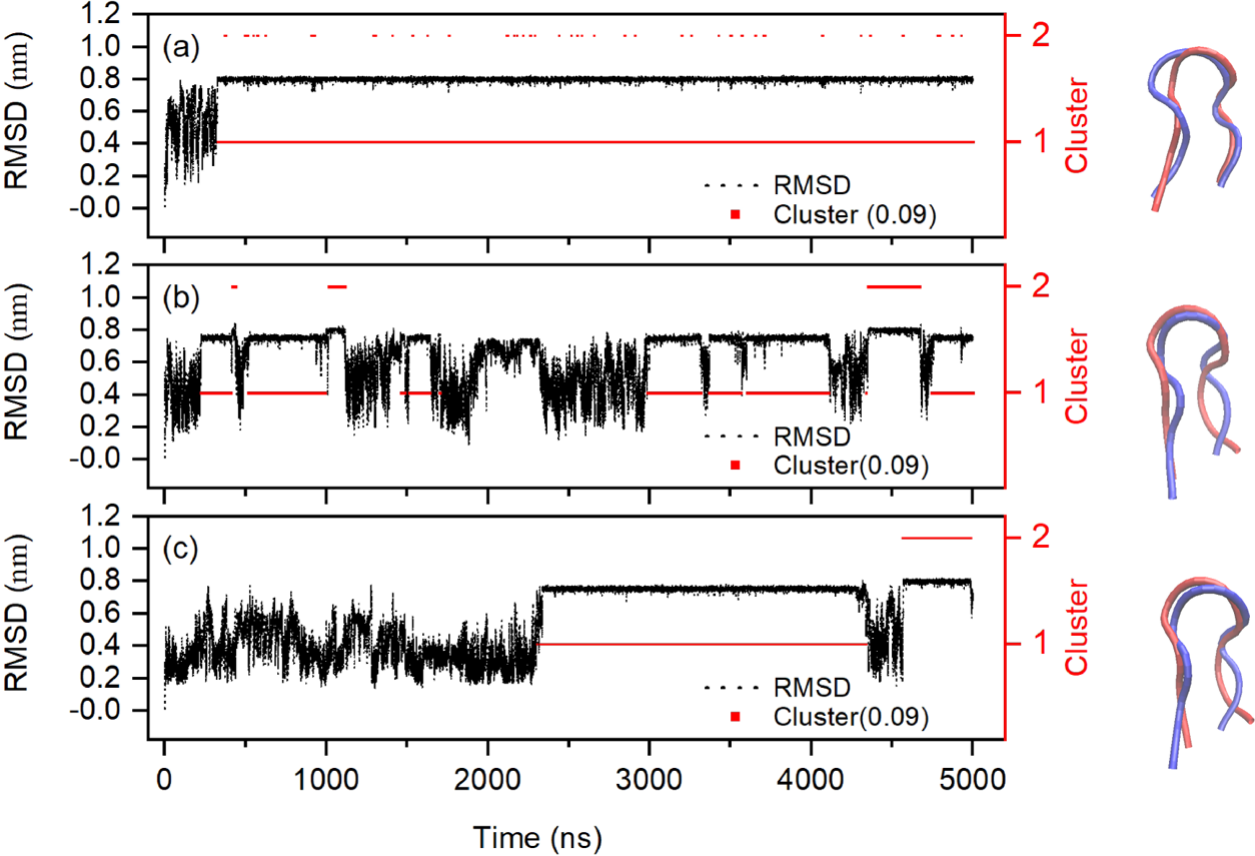
Backbone root-mean-square deviation of chignolin relative to the linear unfolded configuration under different field conditions; structures on the right are the result of 0.9 Å cutoff cluster analysis: cluster 1 (red) and cluster 2 (blue).

As shown in Figure 1, chignolin can fold from a linear unfolding conformation (RMSD = 0 Å, vis-à-vis this linear state) to its well-known “horseshoe-shaped” fold-structure (RMSD > 7 Å) under all three field conditions. In the zero-field case, chignolin can retain the folding structure after the first folding finishes—i.e., for 92.6% of the total simulation time. Under external electric fields (especially under oscillating fields), multiple folding events can be observed, and the folding state accounted for 47.6 and 47.5% of simulation time, under oscillating and static fields, respectively.

For these 5 μs long simulations, it is difficult to identify intuitively all obvious characteristics among conformation-to-conformation transitions across different external-field conditions.^40^ However, some more salient candidates do present themselves, upon further analysis. To derive the representative group of distinct structures during the folding process, we have applied methods for cluster analysis of all of the trajectories using the root-mean-square coordinate deviation of the chignolin’s backbone. Here, the GROMOS cluster-definition method^41^ counts the number of neighbors based on the desired cutoff, taking one structure with the largest number of neighbors with all its neighbors as one cluster and removing this cluster from all 100-ps-spaced trajectory frames—iterating the same algorithm to the rest of the frames, until all of them have been assigned to clusters (or configurational/fold states).

**Figure 2:**
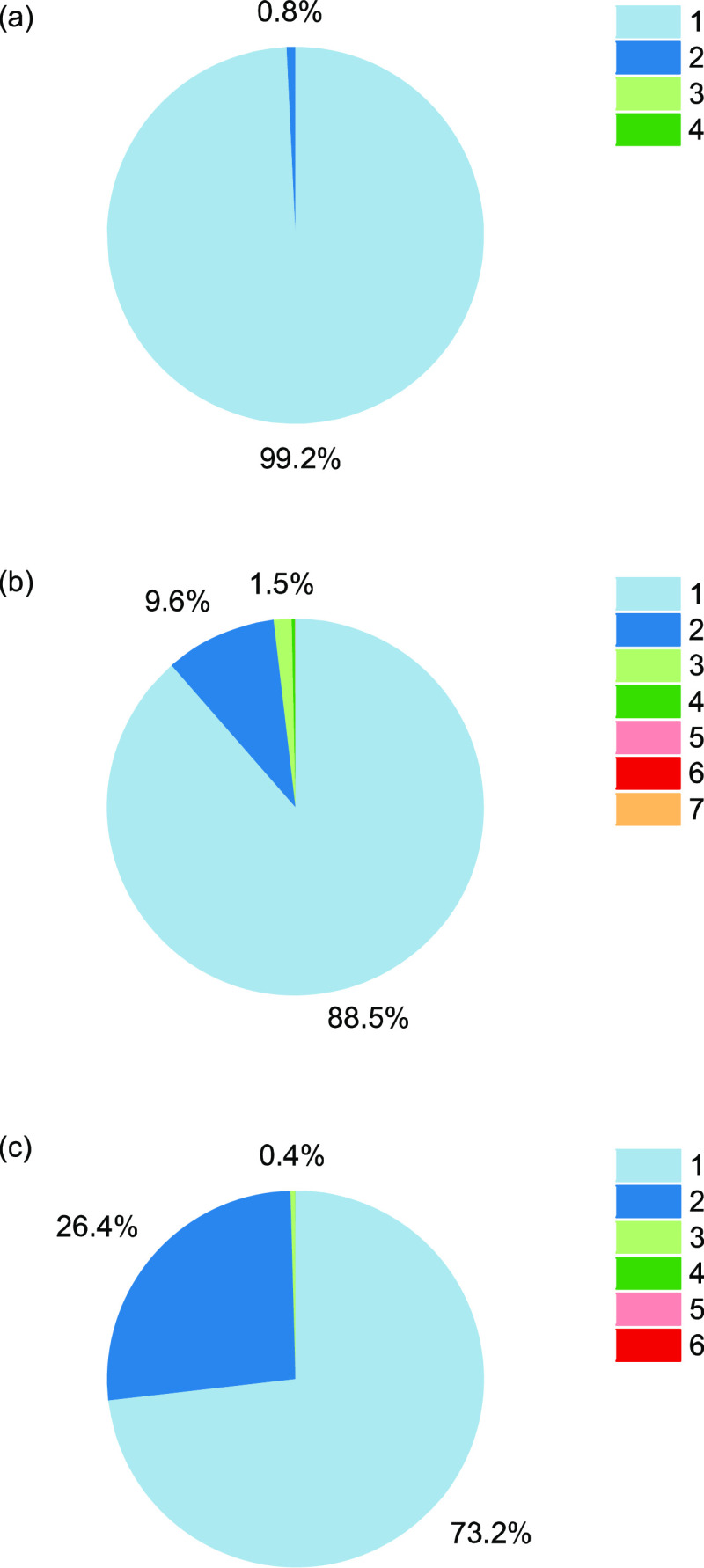
Probability distribution of each state/cluster at (a) zero field, (b) oscillating field, and (c) static field.

**Figure 3:**
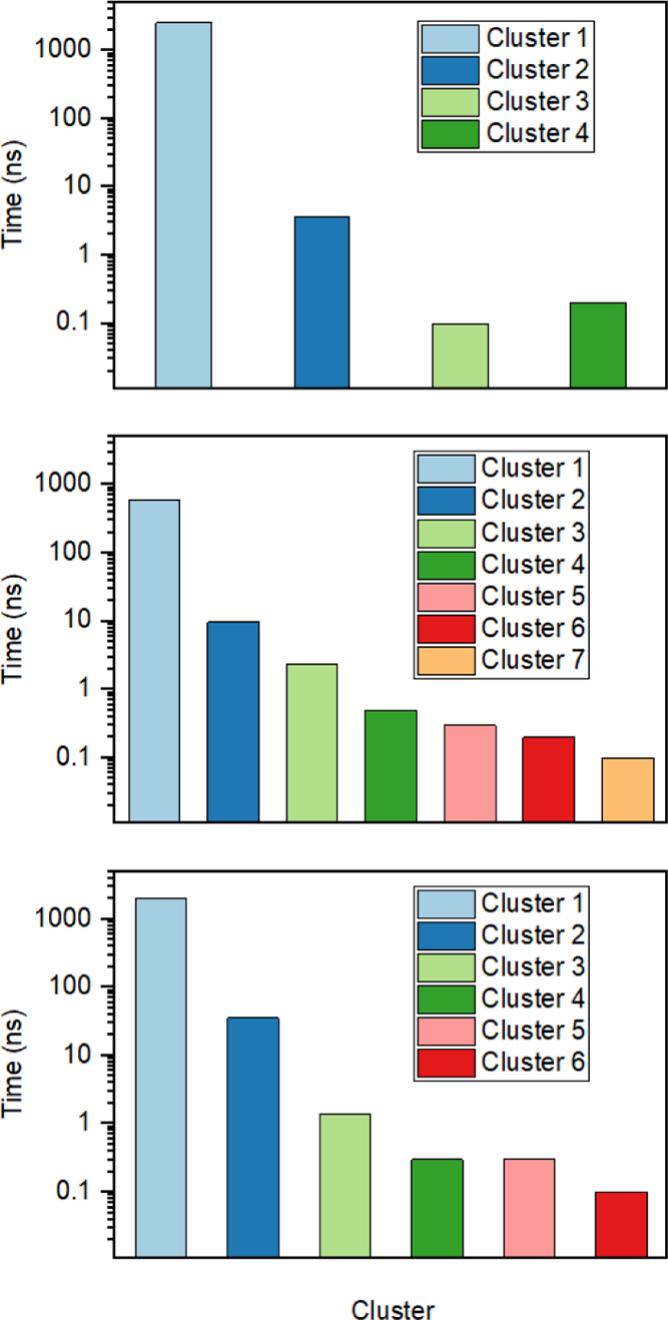
Longest residence times in each fold state for (a) zero field, (b) oscillating field, and (c) static field.

First, a 3.5 Å cutoff was used to obtain all of the possible states throughout all of the trajectory frames; among these clusters, only one cluster accounted for a high population of the total frames under different field conditions (cf. Figure 2). For the zero-field case, there are four groups of clusters, among which the first cluster comprises 99.8% of all structures, which is consistent with the intuitive impression emerging from Figure 1. Since only one folding event occurs under the zero-field case, the third and fourth clusters can be regarded as transition structures from the unfolding structure to the folding structure. Now, in rather stark contrast, for the external-field cases, new clusters were found, implying the existence of new folding paths. Moreover, there are seven and six clusters, respectively, for the oscillating- and static-field cases, and the largest cluster contributes 88.5 and 73.2% for these respective cases. This indicates that chignolin experienced more frequent folding events in the oscillating-field régime, while the static field causes a greater degree of chignolin denaturation over the total 5 μs time-scale. Elaborating on this theme, Figure 3 depicts the longest residence time of each fold configuration sampled over the 5 μs simulations—i.e., the longest dwell times in each state, from entry to exit at either end of the state-to-state transitions. In the zero- and oscillating-field cases, the longest state-residence time occurs for the horseshoe-shaped case, which features more hydrogen bonds between residues. This can be explained by the sustained nature of the field’s strength and direction (by definition) facilitating continued levels of partial dipolar alignment and greater orientational order—in direct competition with underlying thermal motion and sampling. This serves to de-populate the lesser-occupied states/clusters (e.g., no. 7 seen in the oscillating-field case).

**Figure 4:**
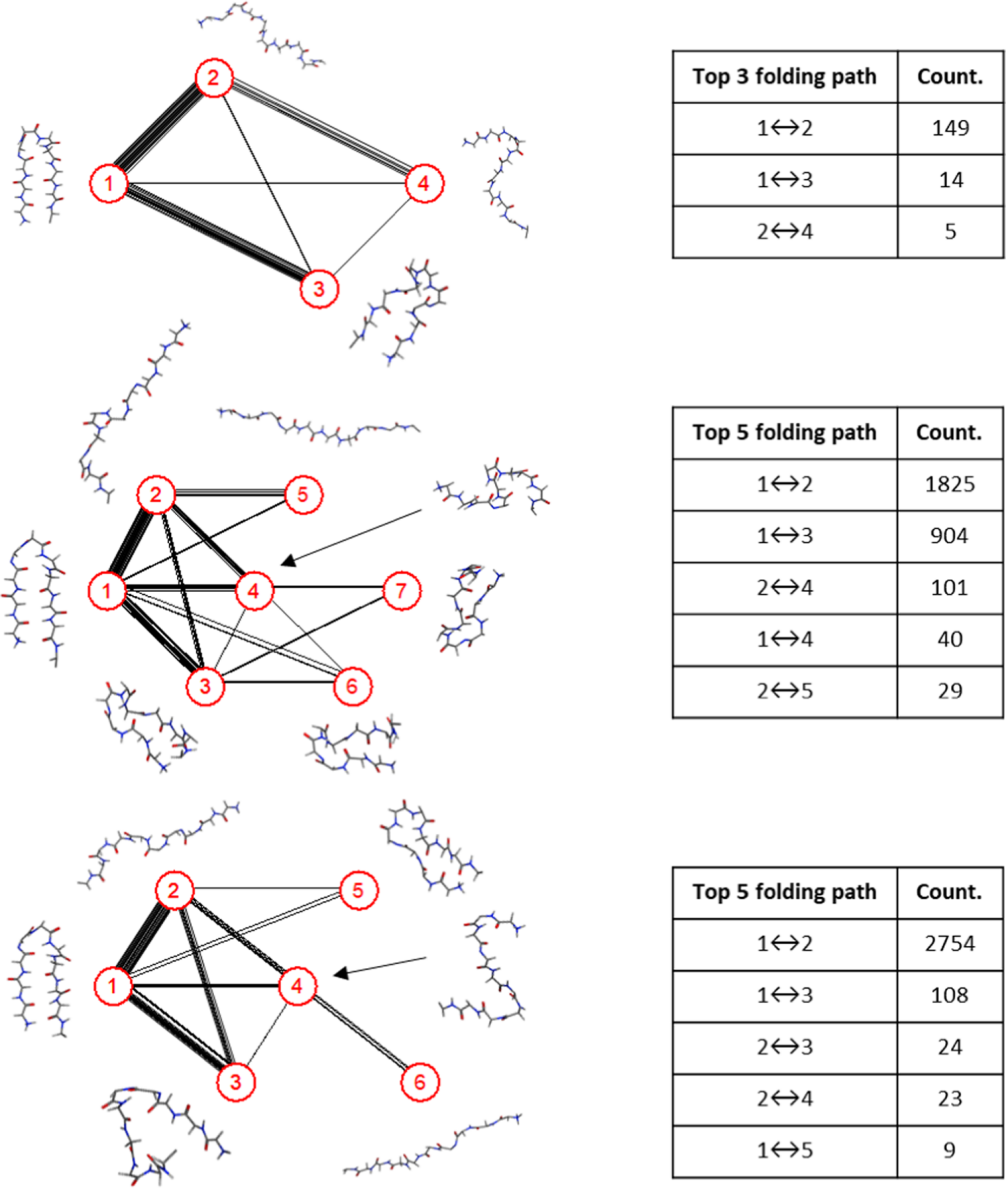
Transition pathways and representative structure of clusters at (a) zero, (b) oscillating, and (c) static fields.

Figure 4 shows the transition pathways between each cluster, and the number of lines indicates the probability of transition. For the zero-field case, all clusters can transform into each other, indicating the inherent disorder of changes in polypeptide structure. Similarly, under external-field conditions, most of these transitions occur among the first four clusters, although some clusters cannot transform between each other directly (e.g., between cluster nos. 5 and 7 under 2.45 GHz). Moreover, compared to oscillating fields, the contribution of the transition between cluster nos. 1 and 2 under static field increases from 1825 to 2754 (i.e., from 62 to 93.6%). The total number of intercluster transition pathways was enumerated as 2945 and 2943 in oscillating and static fields, respectively, indicating the inhibiting effect of the static field on sampling the diversity of chignolin folding conformations and interstate transitions, rather than the aggregate number of such transitions overall.

**Figure 5:**
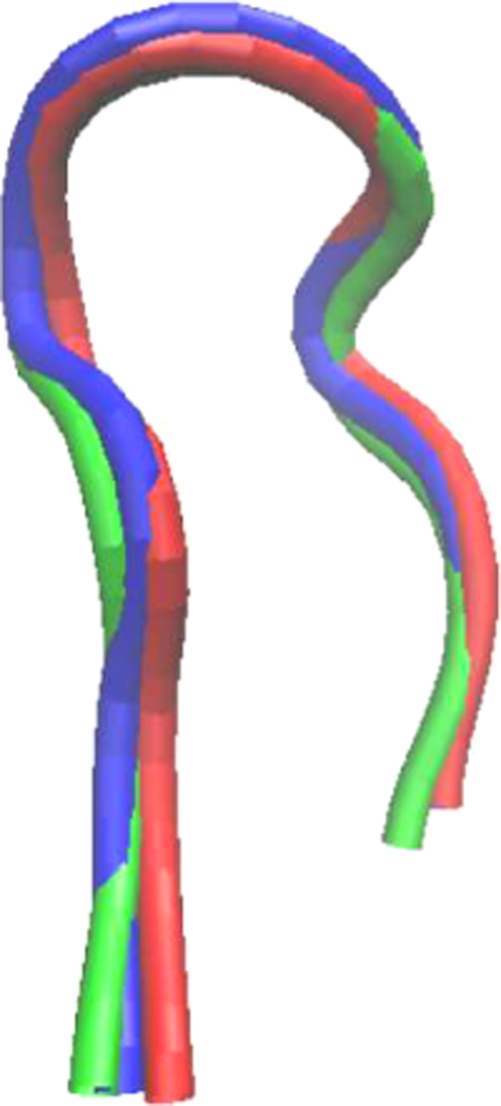
RMSD alignment of represented structures by 0.9 Å cutoff cluster analysis (cluster 1 under zero field: red, cluster 2 under oscillating/static field: blue/green).

To determine more detailed structural configurations, the same cluster analysis was performed with a 0.9 Å cutoff and a large number of clusters were obtained (zero field: 782, oscillating field: 6260, static field: 3917), where the first and second clusters were defined as different stable horseshoe conformations. By aligning the RMSD (cf. Figure 5 and Table S1 in the Supporting Information), cluster 1 under zero field and cluster 2 under oscillating/static field were determined as the native structure, contributing 8.7 and 8.3% of all structures, respectively. Consequently, cluster 1 under both oscillating- and static-field conditions represents the misfolded structure and is composed of 38.9 and 39.2% of all structures, respectively—indicating that they could be more thermally optimized than the native structure under applied external electric fields.

### Hydrophobic Analysis

Hydrophobic interactions have a crucial effect on protein folding, and there is a broad consensus that the folding process of chignolin is initiated in the hydrophobic core.^3,42−44^ Thus, weakening the hydrophobic interaction between the hydrophobic residues Tyr2 and Trp9 (e.g., replacing the Tyr2 with alanine.^42,45^) can affect the stability of folding and misfolding conformations. To investigate how the external field impacts the hydrophobicity of chignolin, signatures of hydrophobic interaction for different clusters were evaluated and compared under different field conditions (cf. Table 1). The cluster featuring the horseshoe-shaped structure (Figure 4) corresponds to the highest average RMSD, and the largest time-averaged number of intraprotein hydrogen bonds—conforming to the thermally accessible native fold. For most clusters, the solvent-accessible surface areas (SASA) are positively correlated with the distance of the Tyr2 and Trp9. But there is no significant difference in the time-averaged number of hydrogen bonds between heavy atoms of Tyr2/Trp9 and water molecules for different clusters since chignolin has only 10 amino acids and the hydrophobic groups cannot be completely buried; clearly, this, in and of itself, cannot constitute a sufficient barrier to suppress the natural hydration process between each residue and water molecules.

**Table 1:** Basic Molecular Information under Different Field Conditions Based on Clusters with a 0.35 nm Cutoff

Zero Field					
cluster	RMSD (nm)	SASA (nm^2^)	distance of Tyr2–Trp9 (nm)	no. of hydrogen bonds (p–p)	no. of hydrogen bonds (w–r29)
1	0.78	7.05	0.55	6.31	6.72
2	0.28	8.37	1.52	1.24	6.76
3	0.6	7.51	0.94	4.4	5.1
4	0.35	8.07	1.53	2	6.17

Oscillating Field					
cluster	RMSD (nm)	SASA (nm^2^)	distance of Tyr2–Trp9 (nm)	no. of hydrogen bonds (p–p)	no. of hydrogen bonds (w–r29)
1	0.67	7.47	0.84	4.63	5.36
2	0.323	8.339	1.603	1.273	6.514
3	0.727	7.059	0.609	4.27	4
4	0.434	7.536	1.243	3.377	5.41
5	0.224	8.6	2.075	0.261	6.61
6	0.683	7.219	0.764	4.545	4.45
7	0.656	7.478	0.905	3	4.33

Static Field					
cluster	RMSD (nm)	SASA (nm^2^)	distance of Tyr2–Trp9 (nm)	no. of hydrogen bonds (p–p)	no. of hydrogen bonds (w–r29)
1	0.656	7.451	0.818	5.158	5.2
2	0.292	8.298	1.649	1.54	6.52
3	0.666	7.924	0.933	2.183	6.36
4	0.46	8.118	1.432	1.115	6.42
5	0.689	7.164	1.024	4.571	5.28
6	0.233	8.721	2.074	0	7.5

Thus, the unnatural behavior of hydrophobic groups under external fields has a strong connection with the change in hydrogen-bond dynamics. Table 2 shows that under applied fields, there is a lower time-averaged number of these two types of hydrogen bond than for the case without field. One explanation is that the electric field accelerates the rearrangement dynamics of the hydrogen-bond network, which was observed in earlier work;^10,11,13,26,28^ indeed, stronger, shorter-duration external-field application and detailed hydrogen-bond dynamic analysis will be discussed in a later section. In addition, electric fields can also affect a protein’s hydrophobicity by changing the motion of specific residues. In this vein, ref (46) has reported that pulsed electric fields can impact the underlying hydrophobicity of proteins by inducing changes in tyrosine vibrational modes and causing more hydrophobic groups inside the protein to be exposed to solvents, which is an evident example of the nonthermal field effect on hydrophobic interaction.

**Table 2:** Basic Molecular Information under Different Field Conditions from 0.5 to 5 μs

scenario	RMSD (nm)	SASA (nm^2^)	distance of Tyr2–Trp9 (nm)	no. of hydrogen bonds (p–p)	no. of hydrogen bonds (w–r29)
zero field	0.79	6.98	0.524	6.56	6.76
oscillating field	0.644	7.522	0.89	4.404	5.41
static field	0.582	7.615	0.992	4.439	5.46

### Free-Energy Landscape

**Figure 6:**
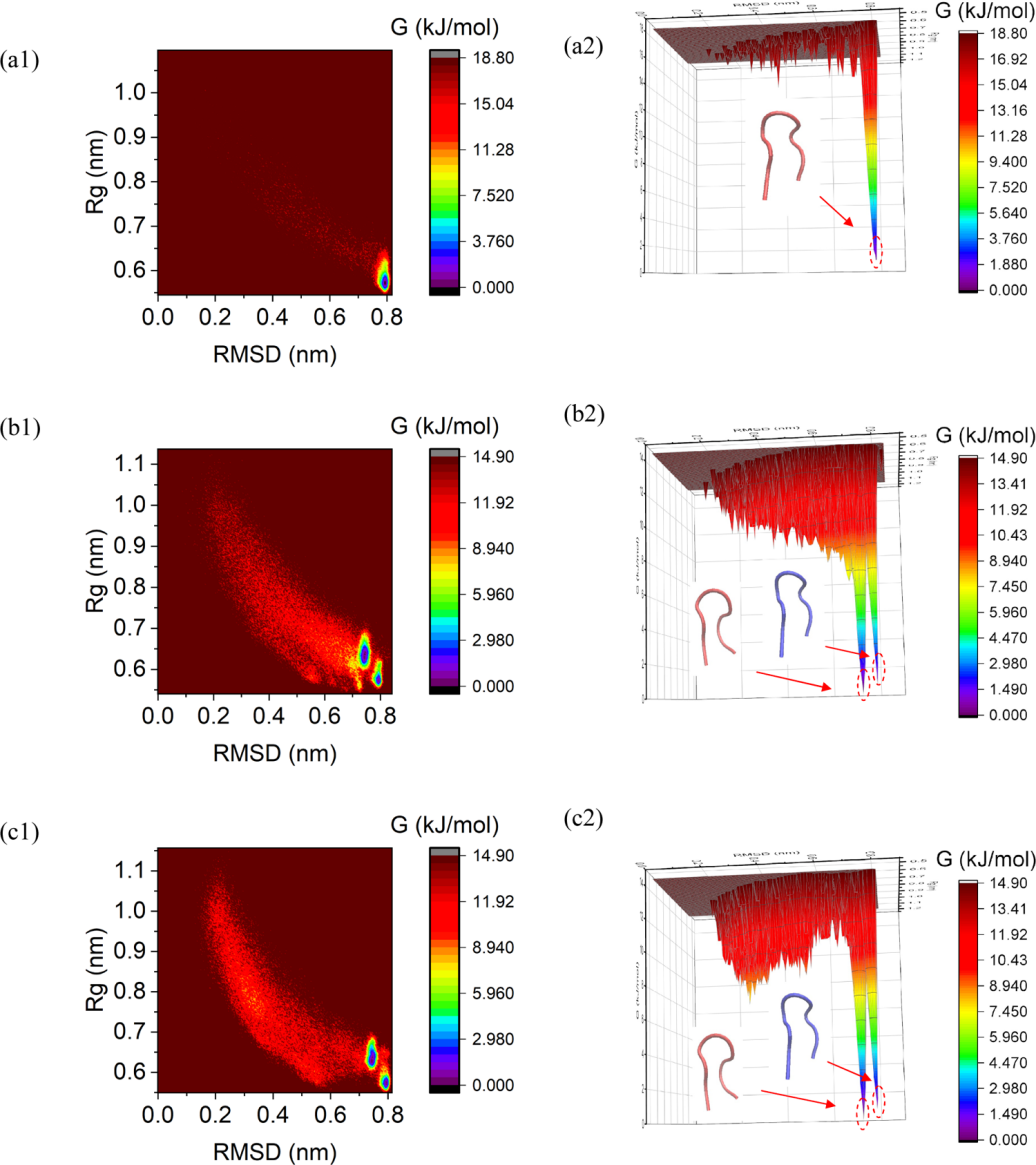
Sampled free-energy landscape for (a1, a2) zero field, (b1, b2) oscillating field, and (c1, c2) static field; left column is the map of the right three-dimensional FEL. For (b2)/(c2), red structures correspond to cluster 1 and blue ones correspond to cluster 2, through the 0.9 Å cutoff cluster analysis.

To obtain further insight into the conformational landscape induced by each field type and strength, the free-energy landscapes (FELs) for all cases were determined by sampling the two-dimensional probability distributions of the chignolin’s radius of gyration (*R*_g_) and its backbone RMSD over the full trajectories (cf. Figure 6). The free energy was characterized using the statistical thermodynamics formulation −*k*_B_*T* log(*N*_*i*_), where *k*_B_ is the Boltzmann constant, *T* is the temperature (300 K), and *N*_*i*_ is the total number of structures contained within a cluster *i* of the two properties. We also corresponded coordinate points in FELs to the results of cluster analysis with the 0.9 Å cutoff, to identify the representative structures in each state and investigate the underlying mechanisms of the folding process.

Under zero-field conditions, there is one energy “bin” near the tiny Rg and large RMSD area (Figure 6a1), corresponding to the native folding configuration (Figure 6a2). Cluster analysis also indicates two clear main states of attraction on each free-energy surface under static and oscillating fields, respectively, which corresponds to the native folding state (Figure 6b2,c2: blue) and the misfolded state (Figure 6b2,c2: red). It is found that there is a high free-energy barrier between the native folding and misfolding states, indicating that these two states cannot transform between each other directly (as was also seen in the pathway analysis of Figure 4). For the oscillating and static cases, there are broad transition states in the 2–7 Å RMSD area: this has the unambiguous implication that the misfolded structure occurs in the early stage of the folding process. In addition, as shown in Figure 6c2, a secondary bin exists in the FEL under static fields, and this area has a higher energy barrier vis-à-vis the folding/misfolding states than under the oscillating field (Figure 6b2). This increase of interstate transition energies arises mechanistically from the landscape-distorting effects of the adoption of larger numbers of hydrogen bonds (Table 2), which also suppresses the diversity and frequency of state-to-state transitions (as witnessed rather dramatically in Figure 2 and by pathway visualization in Figure 4). Under oscillating fields’ entropic action,^47^ there is no population of significant low-energy basins or bins because the field period of 0.42 ns (for the 2.45 GHz fields) tends to be of the general order of inherent (i.e., zero-field) state-to-state shift times and hydrogen-bonding kinetics—which lessens the scope for enthalpy-driven relaxation of chignolin. This dichotomy in the behavior of free-energy-surface shifts has also been witnessed in the case of static- and oscillating-field effects on aquaporin-gating behavior.^48,49^

### Occurrence Frequency of Key Hydrogen Bonds

**Figure 7:**
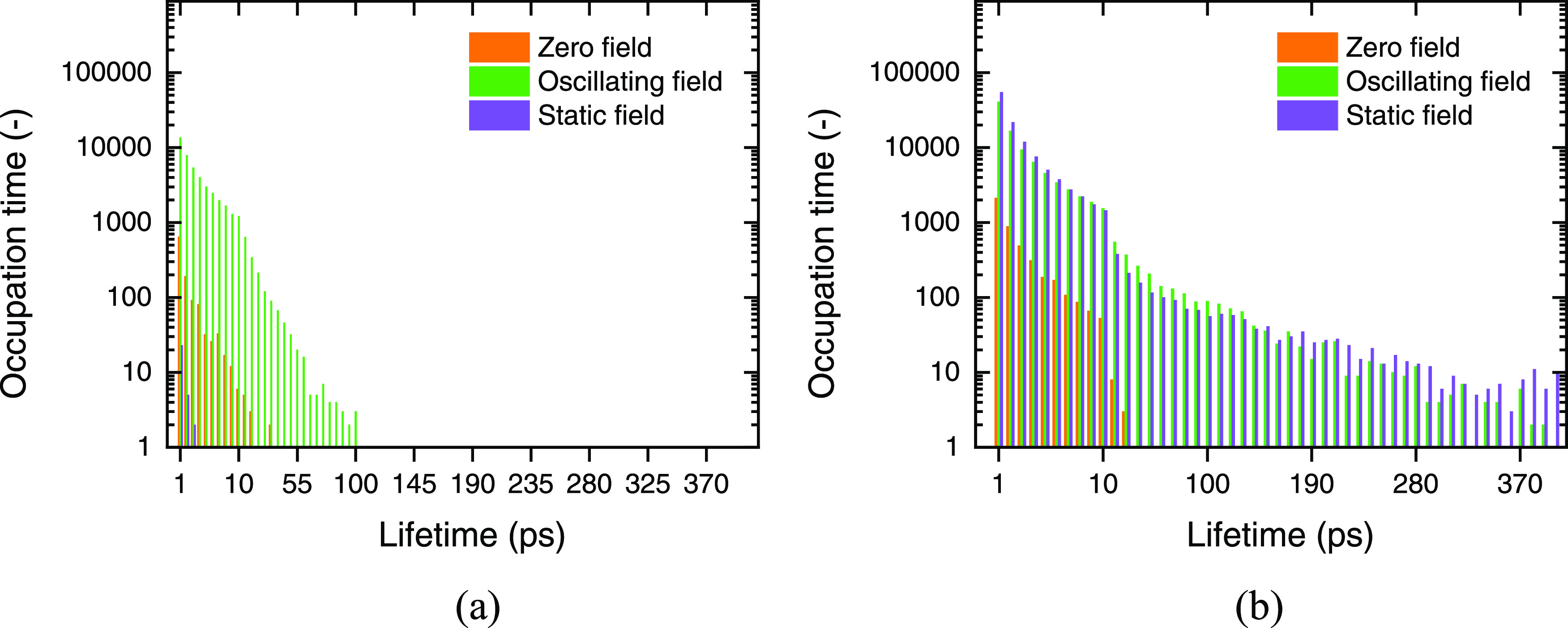
Occupation time distribution of (a) Tyr2N and (b) Trp9N hydrogen bonds under different field conditions.

Intraprotein hydrogen bonds are an important part of maintaining folded structures. During the whole simulation process, almost all skeleton nitrogen atoms and side-chain heavy atoms (N, O) can be used as hydrogen-bond donors to participate in the composition of transition structures and folded structures. To evaluate the perturbation of hydrogen bonds’ reaction dynamics by the applied electric field, we defined the occupation time of hydrogen bonds to count the frame detected over the entire 5-million-frame trajectory, as well as the time of existence between its formation and breakage event, which is the so-called lifetime of a hydrogen bond.^50,51^ Most of these hydrogen bonds are short-lived, only existing for less than 50 ps under all field conditions. Indeed, hydrogen-bond-kinetics analysis of each and every single residue provides a panoply of field perturbations characterized by specific residues (cf. Figure S1, Supporting Information), with a real diversity of field effects. However, it is of particular and considerable note that the hydrophobic residues are especially influenced by the presence of external electric fields: the application of the oscillating field extends immensely the hydrogen-bond lifetimes of Tyr2N and Trp9N, which can be regarded as an important mechanistic consequence of entropic-driven synergy between electric fields and the hydrophobic interaction—with important folding implications, as we have seen already (Figure 7).

**Figure 8:**
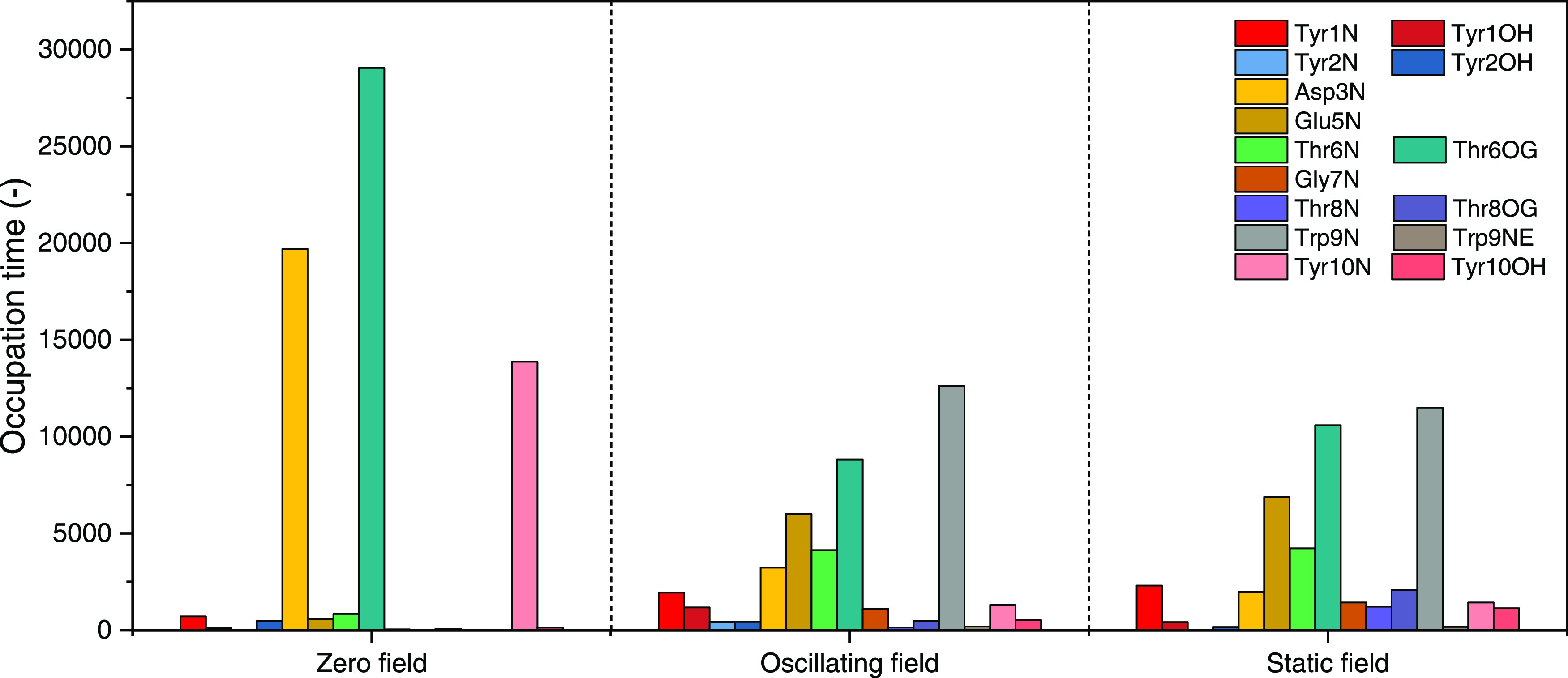
Total occupation time of each residue’s longer-lived hydrogen-bond lifetimes (>50 ps) under different conditions.

Figure 8 provides the occupation time distribution of longer-lifetime hydrogen bonds (>50 ps) and the proton donor is used to represent the group of relative hydrogen bonds. It shows that under zero field, Asp3N, Thr6OG, and Tyr10N contribute more than 95% of all hydrogen-bond occupation time, which play an important role in the native β-hairpin structure of chignolin.^30,52^ The application of external electric fields changes this lifetime distribution significantly: the occupation time of former main hydrogen bonds decreases dramatically, while those of multiple types of hydrogen bonds increase (Glu5N, Thr6N, and Trp9N). These changes may be explained by polar (Thr6) charged (Asp3, Glu5) residues being induced by applied fields, serving to perturb the hydrogen bonds in which their constituent atoms participate. The underlying field effect on hydrophobic interaction (Trp9) mentioned before can also impact the hydrogen-bond lifetimes. Moreover, there is no significant difference in hydrogen-bond lifetimes between oscillating and static fields, since most hydrogen-bond lifetimes are shorter than the period of the 2.45 GHz field (ca. 0.42 ns).

### Ramachandran Analysis

**Figure 9:**
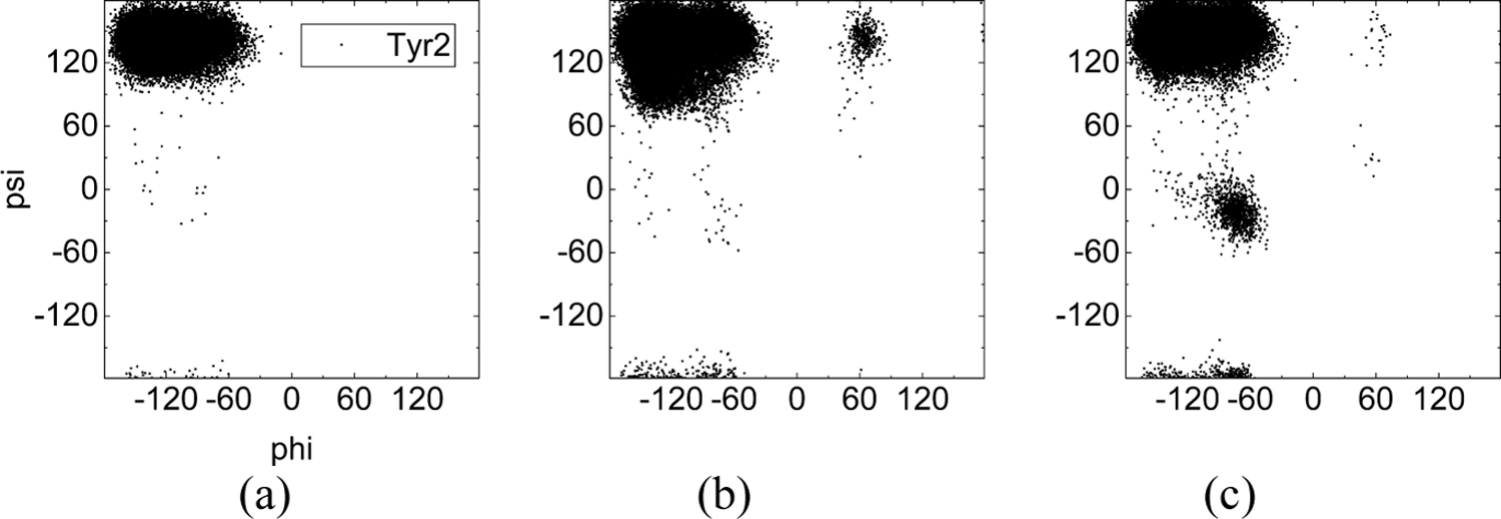
Distribution of backbone torsion angles for Tyr2 for all trajectories under (a) zero field, (b) oscillating field, and (c) static field.

Overall, external electric fields cause a significant change in the ϕ, ψ distribution of chignolin’s residues, which expand underlying structures (cf. Figure S2, Supporting Information). Under the influence of electric fields and hydrophobic interaction, Tyr2 shows a distinctly different response to the oscillating and static field (cf. Figure 9): the change of Tyr2’s torsion-angle distribution arises primarily from the bifurcated behavior of the Pro4 between static and oscillating fields. During the chignolin-folding process, there is frequent hydrophobic contact between Tyr2 and Pro4, by which the rigidity of the N-strand is enhanced, and this serves to reduce the conformational-space options for chignolin.^53^

**Figure 10:**
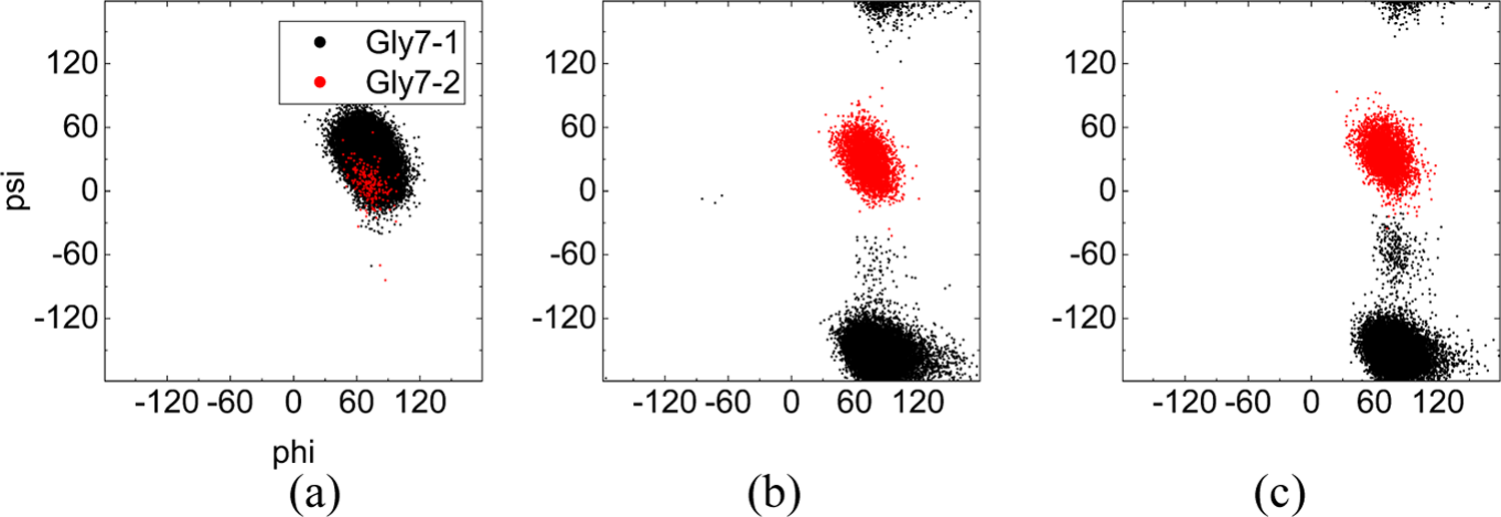
Distribution of backbone torsion angles for Gly7 for clusters 1 and 2 (0.9 Å cutoff) under (a) zero field, (b) oscillating field, and (c) static field.

As the simplest amino acid, glycine has a single hydrogen atom as its side chain, and it plays an important role in the formation of chignolin’s β hairpin structure. Compared with other amino acids, the lack of side chain for glycine allows for a larger number of combinations of ϕ and ψ to be sampled without steric clash, allowing a rotationally symmetric torsional-angle distribution in the Ramachandran plot (cf. Figure. S2).^54,55^ As shown in Figure 10, there was only αL for Gly7 under zero-field conditions, and both αL and βPR structures can be formed under external electric fields, which was the most significant difference between the native (no. 2) and misfolded structures (no. 1).

The differences in the dihedral distribution of the Gly7 residue between the native state (−30 to −90°) and misfolded (−180 to −120°; 150 to −180°) chignolin structure have been widely investigated.^52,56−59^ To investigate the origin of this change in Gly7, Kührová et al.^56^ eliminated the misfolded structure of chignolin by replacing the glycine (Gly7) with lysine. Hatfield et al.^57,58^ reported that increasing temperature (333 and 363 K) expanded the distributions in the left-handed helix region area of Gly7, and other solvents, such as MeOH and dimethyl sulfoxide, can limit this transition in the native state area. The primary difference vis-à-vis the previous simulation lies in a “transition” area in the −90 to 30° region induced by static fields, which can be seen when combined with the free-energy landscape (cf. Figure 6c2) and other residues in the same structural range, with only a limited degree of angle shift occurring (cf. Figure S3).

## Conclusions

NEMD simulations were performed to determine the effect of both static and alternating externally applied electric fields on solvated chignolin’s exploration of its room-temperature folding-transition dynamics and states. A key focus was on how external fields alter folding pathways athermally, using thermostatted NEMD. Dramatically different, bifurcated behavior of incipient chignolin-folding processes was seen between both static and oscillating electric fields: in the former case, the number of states was reduced, with fewer, well-defined state-to-state transitions, while in the latter case, state populations diversified with an attendant acceleration of state-hopping folding kinetics. This reflects the fundamentally different reaction of intraprotein hydrogen-bonding characteristics to static and oscillating electric fields (borne of differing dipole alignment and the local translational response of charged residues)^48,49^—with shifting of folding-energy landscapes by static fields to have a more rugged, spaced-out terrain,^50,51^ and a relatively smooth transition area out of such evidence in the case of oscillating fields, to inhibit folding-state relaxation into either of the two natural energy bins (with natural thermal “flitting”—or folding—in between).

The use of linear-response, lower-intensity external fields has been important in the present study to allow for folding-phenomena sampling by fully deterministic MD (even in the field-driven, out-of-equilibrium cases via NEMD). This has allowed for mechanistic disentangling of the external-field effects as much as possible from the inherently complex underlying dynamics of protein motion, as well as its underlying structural and thermodynamic behavior—seeing the dramatically different static- and oscillating-field effects at play. In future studies, biased sampling may also be used for time-independent external fields to probe shifting of free-energy landscapes and these terrains’ features^26,48^ should there be a desire to probe lower-intensity fields down toward more everyday intensities, well below the dielectric-breakdown threshold, calling for accurate electrostatics.^60,61^ Application of higher-frequency oscillating fields will be essential to investigate the effect of alternating fields on hydrogen-bond dynamics inside the protein.

## Supporting Information Available

RMSD information (Table S1, cluster-to-cluster), hydrogen-bond numbering (Table S2) and occupation times (Figure S1), Ramachandran plots (Figures S2 and S3), and dipole analysis for chignolin (Figures S5 and S6) and water (Figure S4) based on *x*-component dipole alignment (PDF)
