# Supplementary material for: Effects of Externally Applied Electric Fields on the Manipulation of Solvated-Chignolin Folding: Static- versus Alternating-Field Dichotomy at Play
Source: J Phys Chem B. 2022 Jan 10;126(2):376–86. doi: 10.1021/acs.jpcb.1c06857 (PMC8785190; doi:10.1021/acs.jpcb.1c06857)
Supplement: Supplementary file 1 — jp1c06857_si_001.pdf [file jp1c06857_si_001.pdf]

Effects of Externally-Applied Electric Fields on Manipulation of Solvated Chignolin Folding: Static- versus Alternating-Field Dichotomy at Play

HaoLun Wu[1], Mohammad Reza Ghaani[1*], Zdeněk Futera[2] and Niall J. English[1*]

1.School of Chemical & Bioprocess Engineering, University College Dublin, Belfield, Dublin 4, Ireland

2. Faculty of Science, University of South Bohemia, České Budějovice, Czech Republic

*Corresponding authors: mohammad.ghaani@ucd.ie (MRG); niall.english@ucd.ie (NJE)

Supplemental Information

Table S1. RMSD (Å) between cluster and cluster (a: cluster 1, b: cluster 2) under different conditions

	Zero field (a)	Oscillating field (a)	Static field (a)
Zero field (b)	0.1834	0.4053	0.4258
Oscillating field (b)	**0.0963**	0.3934	0.4149
Static field (b)	**0.1507**	0.3925	0.4068

	Zero field (a)	Oscillating field (a)	Static field (a)
Zero field (a)	0	0.4039	0.4237
Oscillating field (a)		0	**0.0895**
Static field (a)			0

	Zero field (b)	Oscillating field (b)	Static field (b)
Zero field (b)	0	0.1797	0.2278
Oscillating field (b)		0	0.1718
Static field (b)			0

Table S2. Number of hydrogen bond' acceptors based on residues under different field conditions.

	Zero field	Oscillating field	Static field
Tyr1N-	11	17	12
Tyr1OH-	16	25	25
Tyr2N-	10	14	4
Tyr2OH-	19	20	20
Asp3N-	11	13	13
Glu5N-	10	14	10
Thr6N-	10	14	10
Thr6OG-	18	22	21
Gly7N-	12	15	14
Thr8N-	10	13	14
Thr8OG-	18	20	19
Trp9N-	4	10	6
Trp9NE-	15	15	15
Tyr10N-	4	8	7
Tyr10OH-	10	12	12

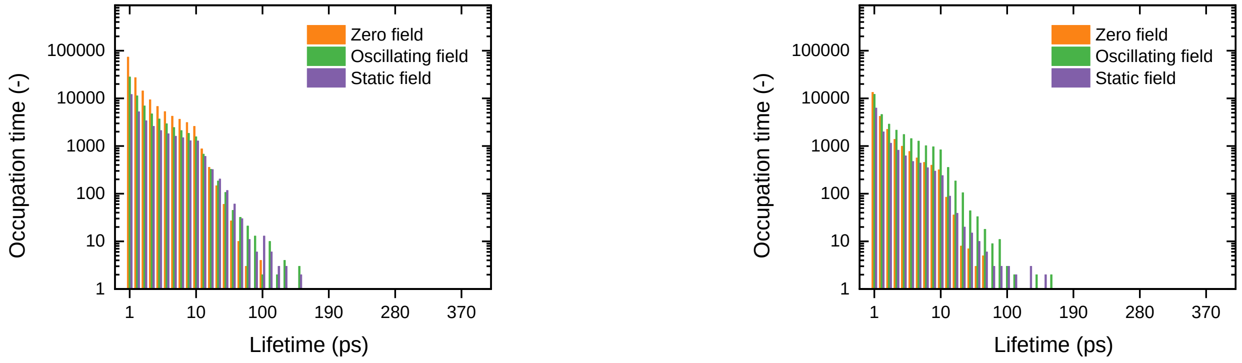

Tyr1N- and Tyr1OH-

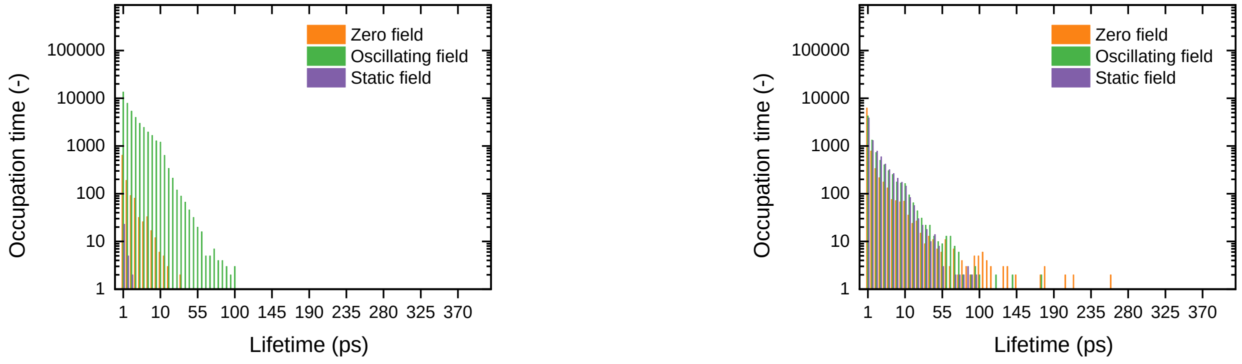

Tyr2N- and Tyr2OH-

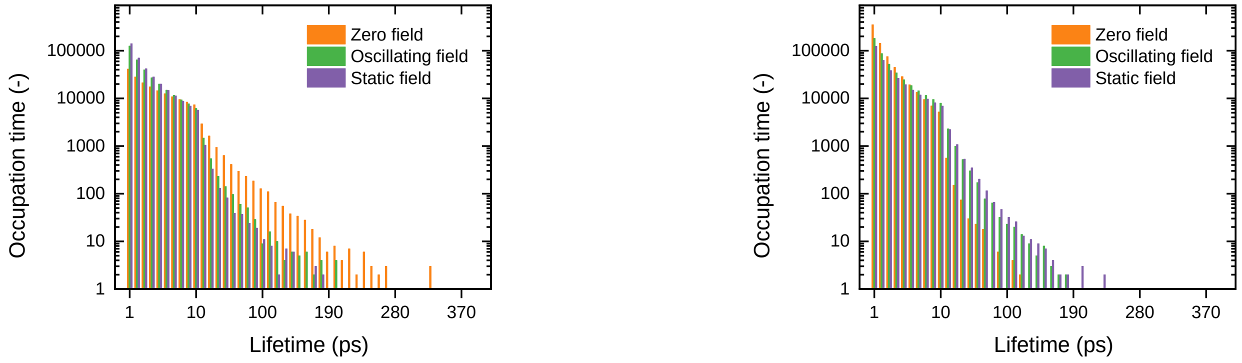

Asp3N- and Glu5N-

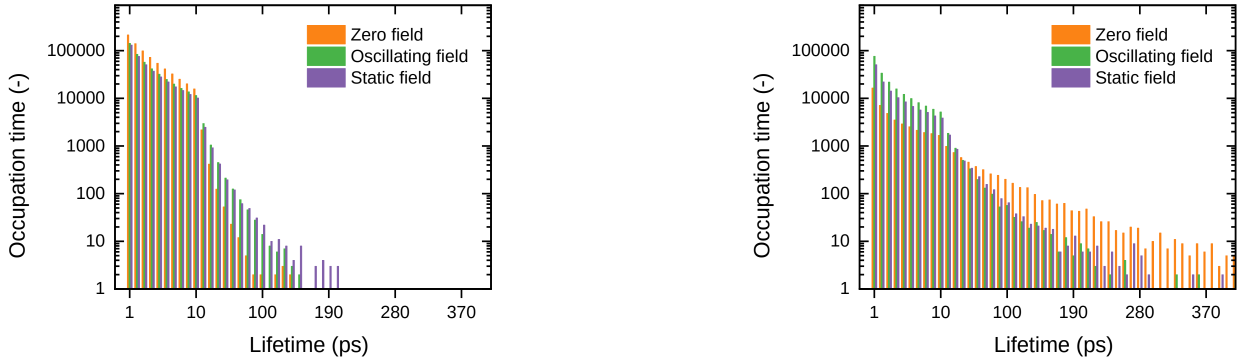

Thr6N- and Thr6OG-

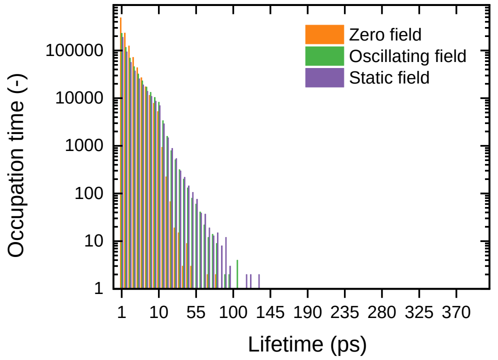

Occurrence distribution of Gly7N-

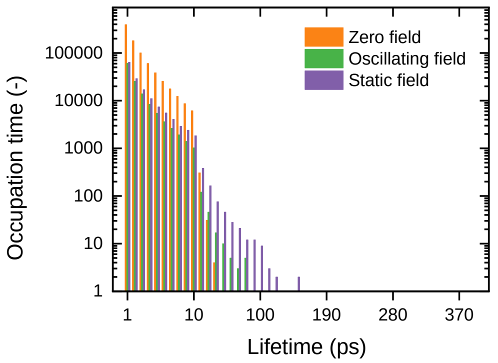

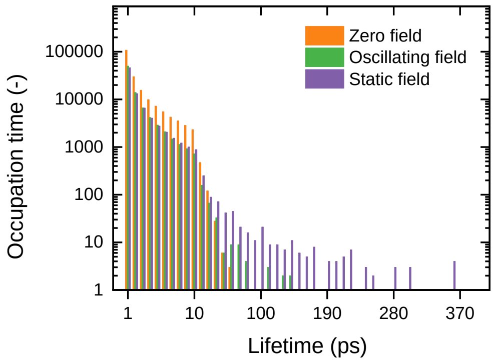

Occurrence distribution of Thr8N- and Thr8OG-

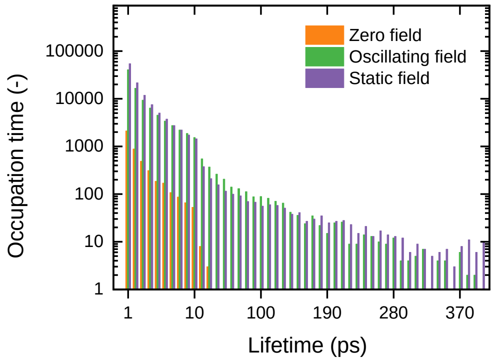

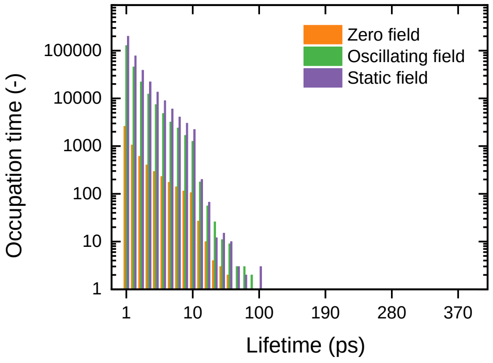

Occurrence distribution of Trp9N- and Trp9NE-

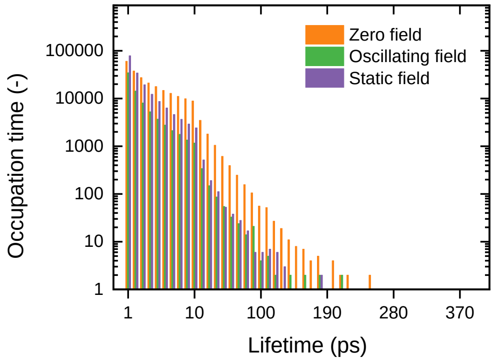

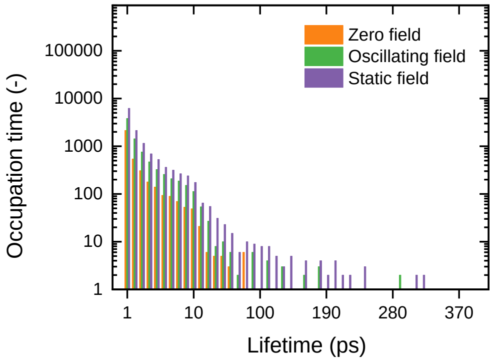

Occurrence distribution of Tyr10N- and Tyr10OH-

Figure S1. Occupation-time distribution of each residues' hydrogen bonds in different field conditions.

Zero field Osicillating field Static field

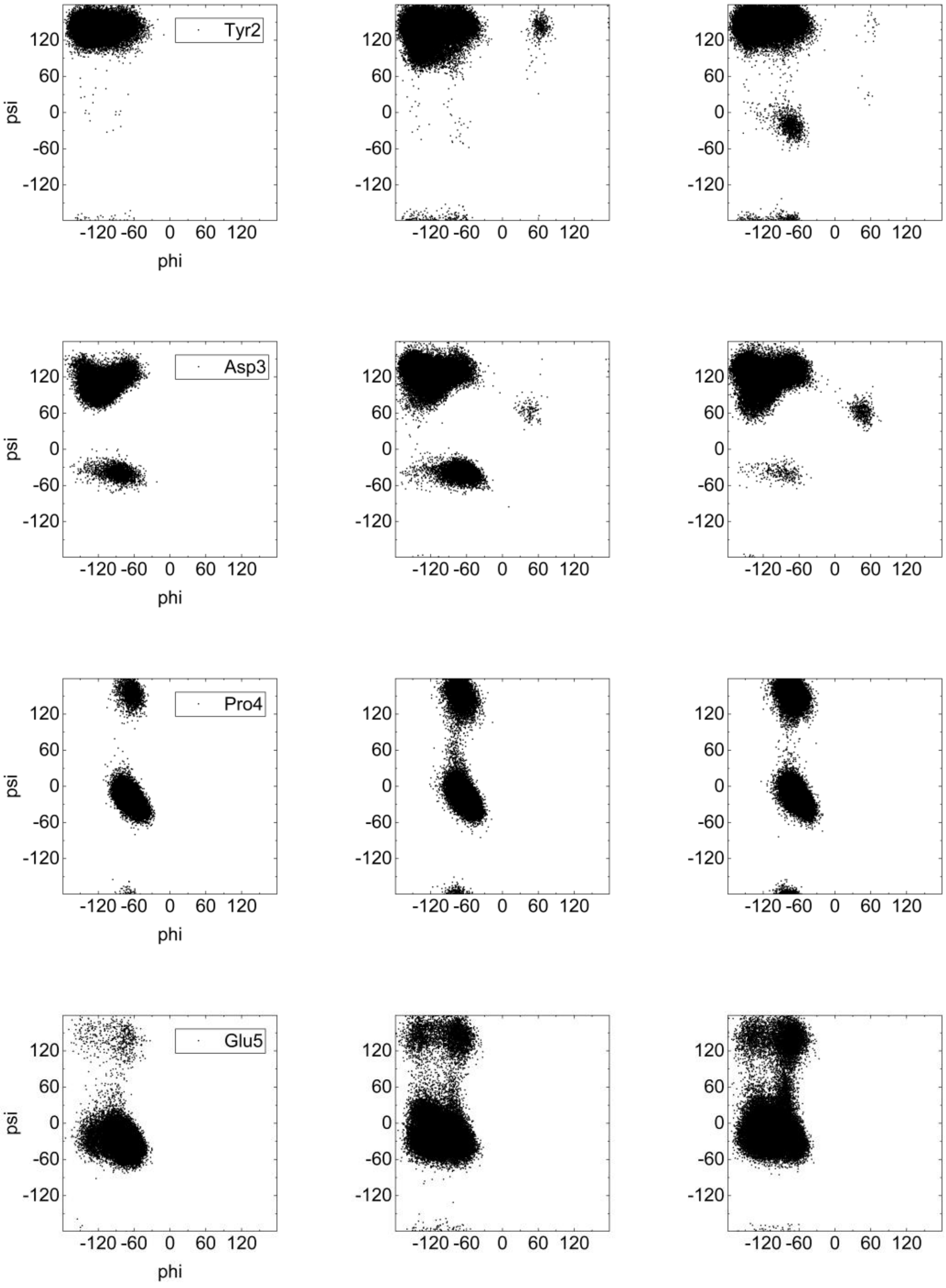

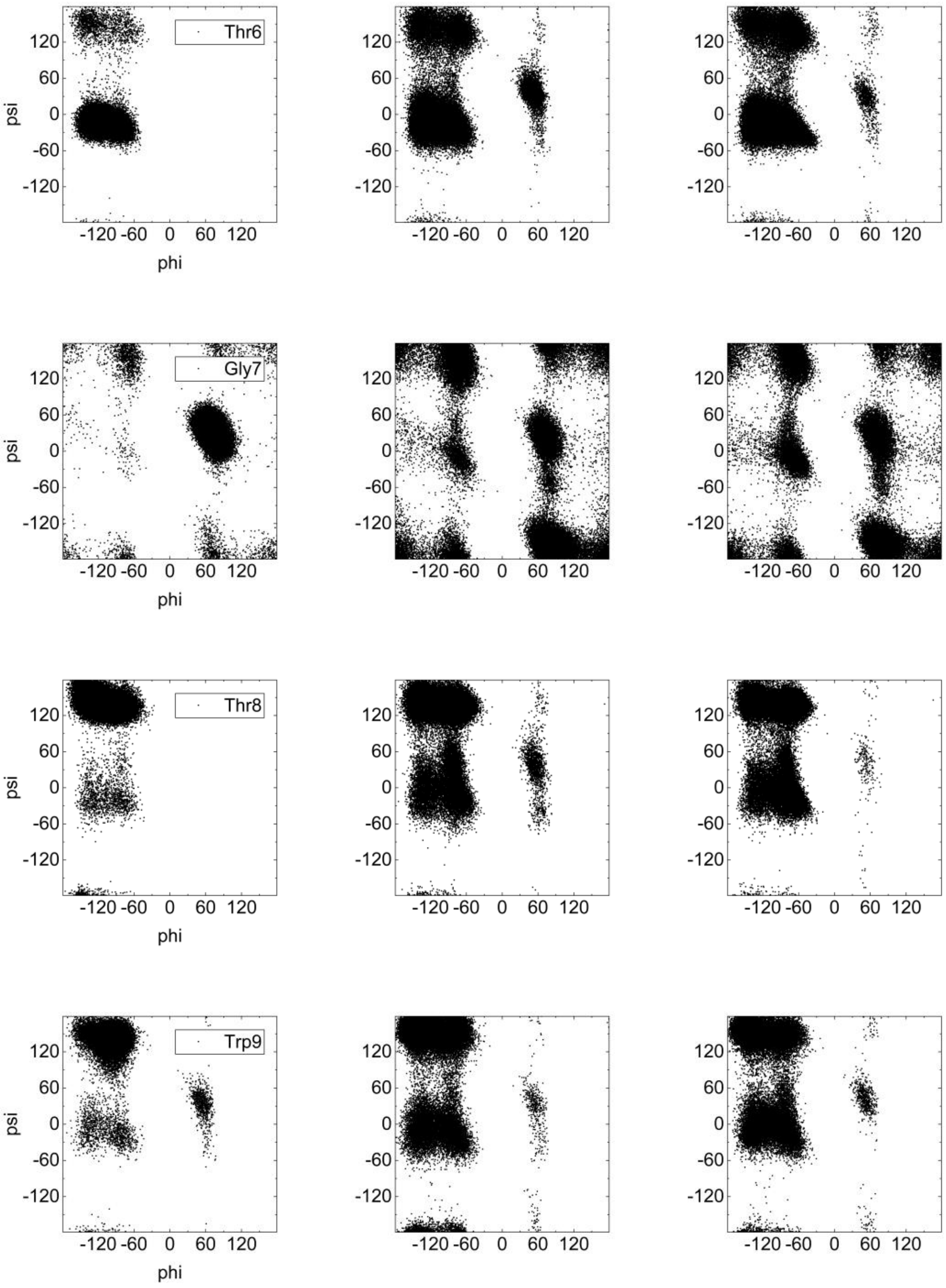

Figure S2. Ramachandran plots under various field conditions.

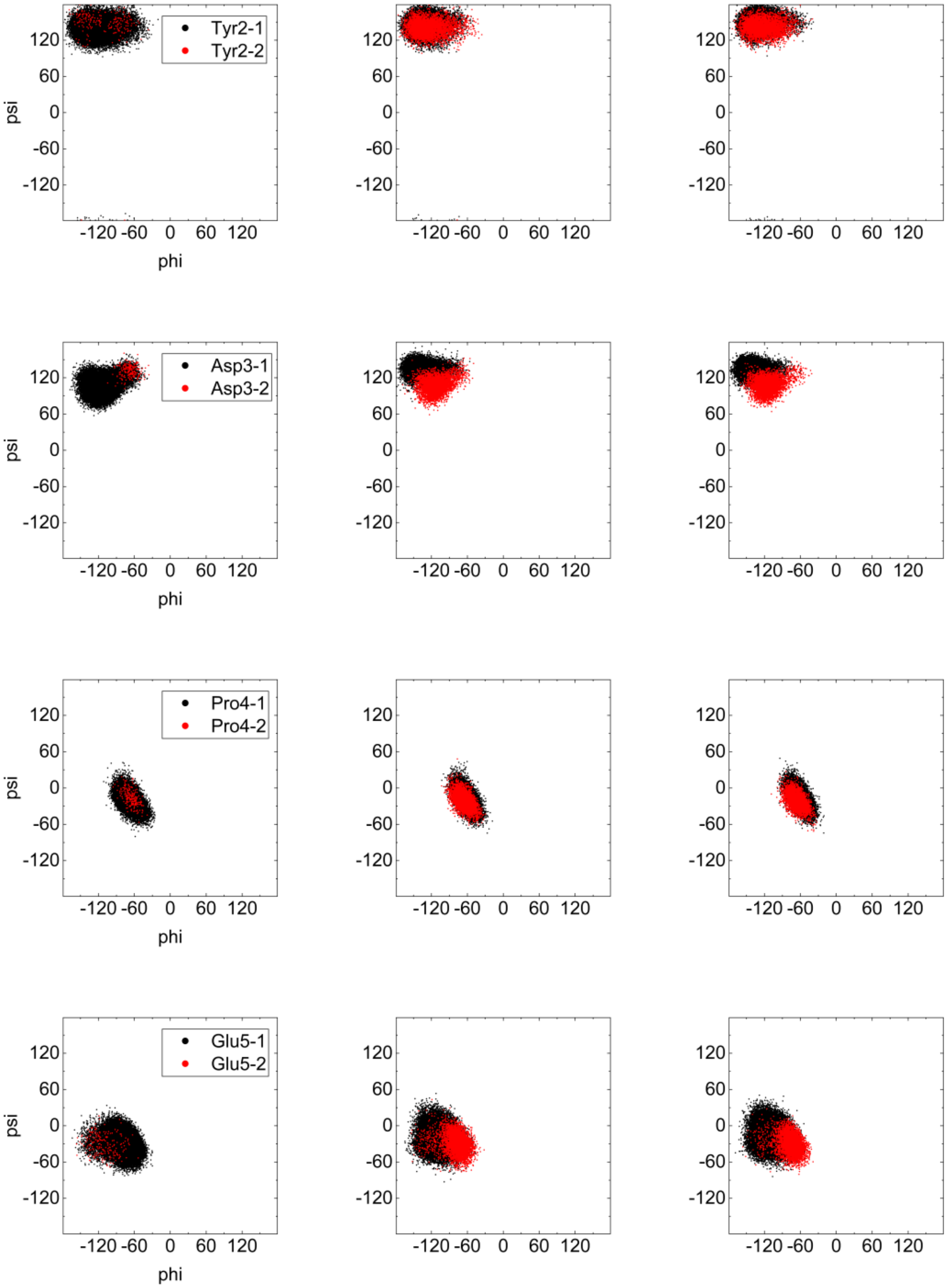
Tyr2-1
Tyr2-2
Asp3-1
Asp3-2
Pro4-1
Pro4-2
Glu5-1
Glu5-2
psi
phi
120
60
0
-60
-120
-120 -60 0 60 120

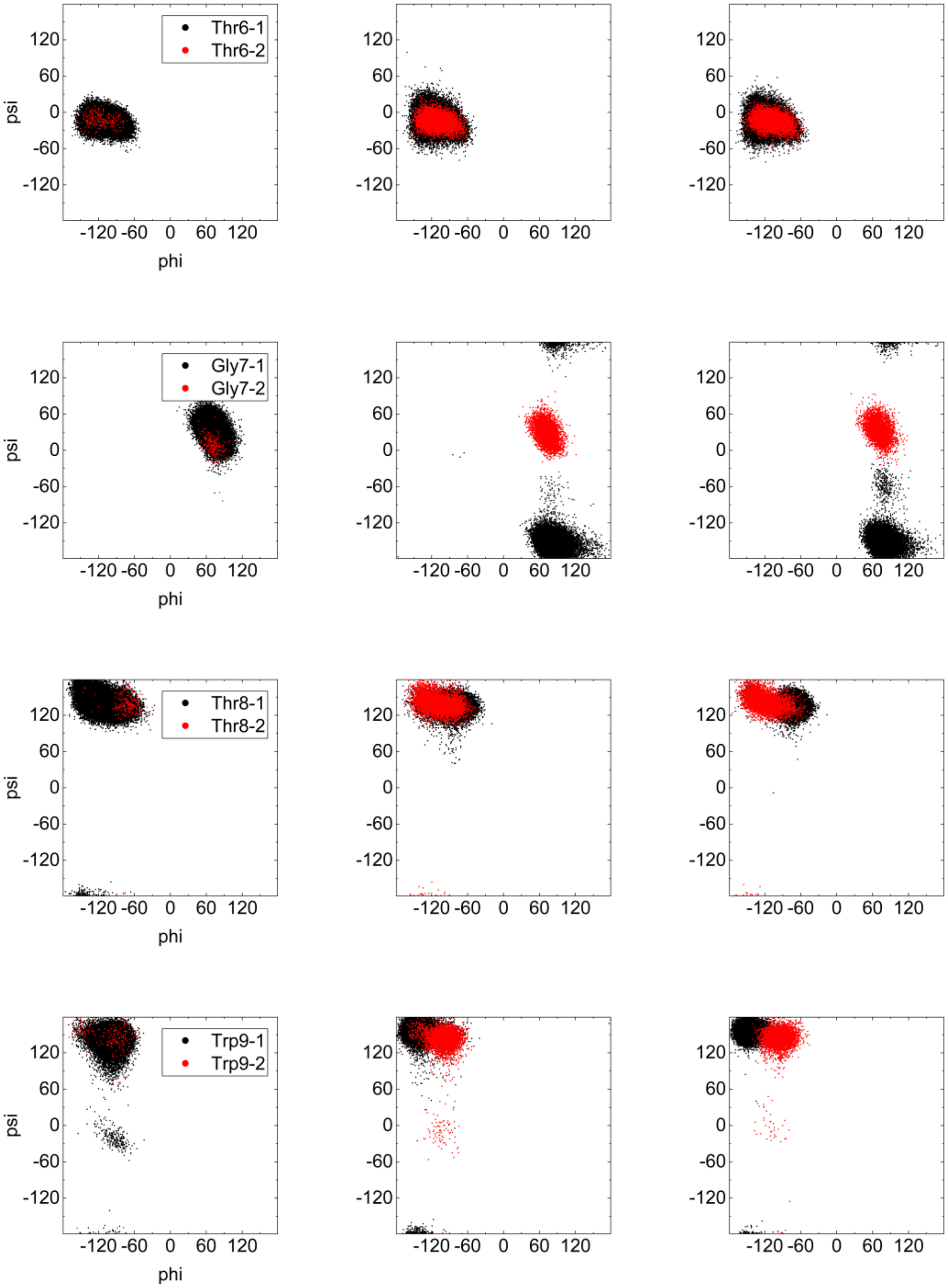

Figure S3. Angle shifts in Ramachandran plots between the first and second cluster under various fields.

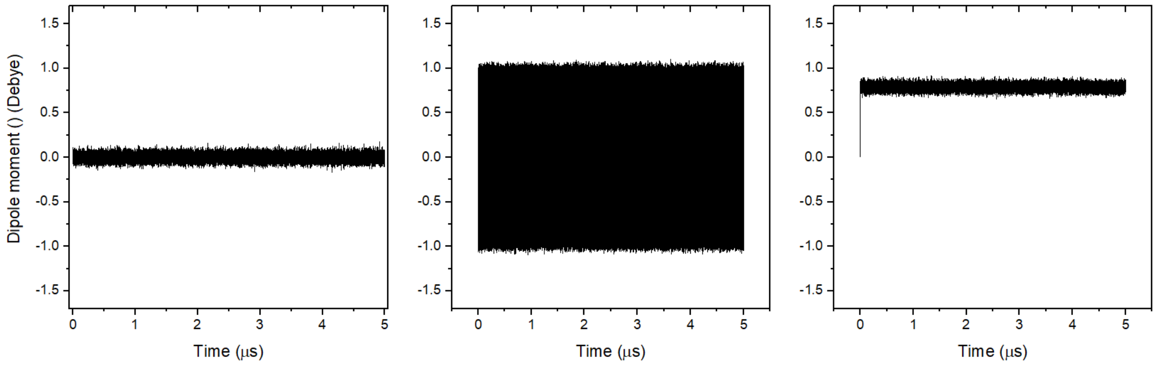

(a) (b) (c)

Figure S4. x-component of the average dipole moment for water molecules in (a) zero field (b) oscillating field (c) static field.

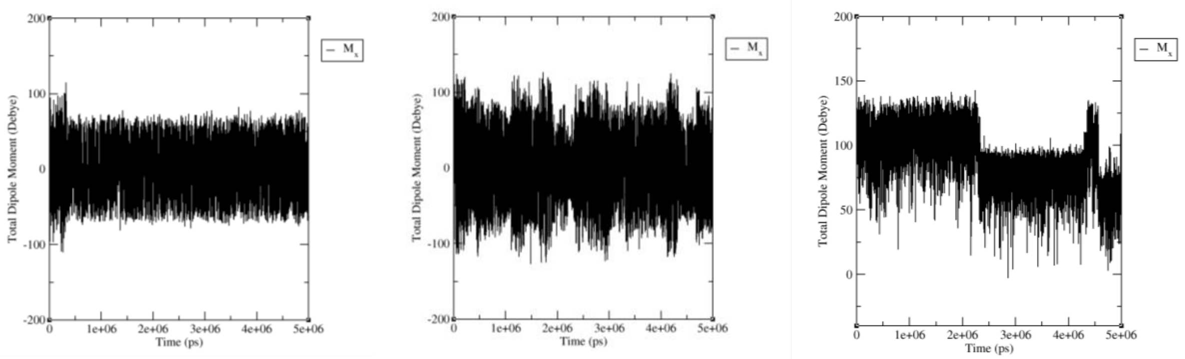

(a) (b) (c)

Figure S5. x-component of the dipole moment for chignolin in (a) zero field (b) oscillating field (c) static field.

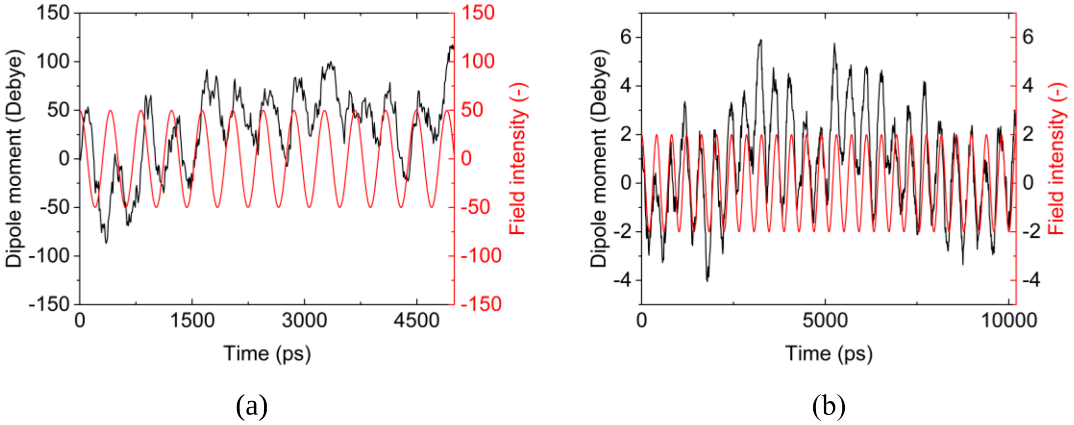

Figure S6. x-component of the dipole moment for chignolin in oscillating field for the initial 5000ps (a) and the average of 10200-ps sampling for the whole simulation (b), where the black line represents the dipole moment of chignolin, and the red line represents the intensity of oscillating field (normalized for clear visualizing).
